# Trajectories in health in late midlife: disparities persist across racial/ethnic groups in US

**DOI:** 10.1093/geroni/igaa057.3371

**Published:** 2020-12-16

**Authors:** Adit Doza, Gail Jensen Summers, Wassim Tarraf

**Affiliations:** Wayne State University, Detroit, Michigan, United States

## Abstract

Introduction: Recent research has revealed that during late midlife, Hispanics experience a lower mortality relative to non-Hispanic Whites (Whites), whereas non-Hispanic Blacks (Blacks) experience a higher mortality relative to Whites. Much less is known about whether there are also racial/ethnic disparities in patterns of how overall health changes during this period, and if so, whether personal lifestyle or economic characteristics that can explain it. We examine these important issues. Methods: Longitudinal and nationally representative data on adults ages 50-64 from the Health and Retirement Study are used for our analysis. Latent class discrete time and growth curve modeling are implemented to identify different types of “trajectories” in self-rated health and mortality across racial/ethnic groups and three distinct time periods, 1998-2004, 2004-2010, and 2010-2016. Additionally, multinomial logit models are estimated to determine whether Hispanics or Blacks experience different trajectory profiles than Whites do, and how different characteristics at baseline affect one’s own trajectory. Results: We identify five types of trajectories: “Poor-Health with Rapid Mortality,” “Sustained Poor-Health,” “Moderate-Health Back and Forth,” “Good-Health with Rapid Mortality,” and “Sustained Good-Health.” These specific types persist even after controlling for multiple health determinants. Discussion: We find significant and persistent differences across racial/ethnic groups in how health changes during late midlife. After controlling for a wide range of health determinants, Blacks and Hispanics are less likely than Whites to experience “Sustained Good-Health” and more likely to experience “Sustained Poor-Health” across all three time periods. Blacks are also consistently more likely to experience “Poor-Health with Rapid Mortality.”

